# Supplementary material for: Obstetrics care in Indonesia: Determinants of maternal mortality and stillbirth rates
Source: PLoS One. 2024 Jul 5;19(7):e0303590. doi: 10.1371/journal.pone.0303590 (PMC11226051; doi:10.1371/journal.pone.0303590)
Supplement: S1 File — (DOCX) [file pone.0303590.s001.docx]

Obstetrics care in Indonesia : Determinants of maternal mortality and stillbirth rates

Supplementary file S1

# Description of data extraction, stratification and weighing

Participations data and services data were assumed to be highly correlated at the family level, which is the legal background of JKN registration rules. Therefore, the sampling process used the family as the smallest sampling unit, not individual participants. According to the reimbursement structure the health service data can be distinguished between FKTP for primary healthcare (PHC) and FKRTL for advanced care and referral (AHC).

Due to restrictions of data protection the entire dataset was not available for the analysis and a representative and randomly chosen sample was obtained:

*Stratified random sampling of health service data*

1. Preparation of a family sampling frame:
   The sampling framework was a family master list containing all sampling units, namely the participant's family (73,441,160 lines) where each row represented one family.
2. The strata used in sampling were based on a combination of two variables, namely FKTP (as many as 22,024 different options, representing primary healthcare, referred as PHC) and three family categories that were determined as:
3. Category 1: families whose members have never received health services
4. Category 2: families that have participants who have received health services in FKTP (= PHC)
5. Category 3: families that have participants who have received health services in FKTP (= PHC) and FKRTL (= AHC)

If all available FKTPs were used for family members from all three categories, this resulted in 3 x 22,024 strata (66,072 potential strata).

1. Selecting a sample of families for extraction of health service data using the stratified random sampling method:
   Each strata was randomly chosen for N=10 families, meaning that the strata with more than 10 families were only taken by 10 families, whereas if the strata size was not more than 10 families, all families in the strata were sampled. This process resulted in a sample size of 586,969 families.
   Using this family selection list the filtering process was applied at the complete data (health service master file) to retrieve data based on the selected sample.
2. Participant sample data were obtained from the complete data (health service master file) of participants through the family code selected in step number 3 producing a total of 1,697,452 health service data of individual participants.

*Determination of Sample Weight*

The individual weight of the results of sampling was needed for correction of selection process that resulted in disproportions between sample dataset and population. This was corrected by determination of sample weights for each individual selected participant. Before calculating individual weights the family weight was determined first. Subsequently, individual weights were calculated by dividing the family weight by the number of family members. The distribution of individual sample characteristics now represented the characteristics of respective individual population.

*Selected health service data for evaluation*

- 1,733,759 PHC service visits reimbursed by capitation (~90% completeness of the dataset)
- 114,820 PHC services reimbursed by non-capitation
- 911,101 AHC services

A variable 'Weight' was provided in the membership data file (see variable list in suppl. table 2). Analysis was carried out with weighting for all health service data so that the results of the evaluation represented the entire JKN member structure.
