# Supplementary material for: Obstetrics care in Indonesia: Determinants of maternal mortality and stillbirth rates
Source: PLoS One. 2024 Jul 5;19(7):e0303590. doi: 10.1371/journal.pone.0303590 (PMC11226051; doi:10.1371/journal.pone.0303590)
Supplement: S2 File — (PDF) [file pone.0303590.s002.pdf]

ICD10 codes used in capitation based reimburesement (outpatient setting)

O10	Prenatal complications	O10 Pre-existing hypertension complicating pregnancy, childbirth and the puerperium
O11	Prenatal complications	O11 Pre-eclampsia superimposed on chronic hypertension
O12	Prenatal complications	O12 Gestational [pregnancy-induced] oedema and proteinuria without hypertension
O13	Prenatal complications	O13 Gestational [pregnancy-induced] hypertension
O14	Prenatal complications	O14 Pre-eclampsia
O15	Prenatal complications	O15 Eclampsia
O16	Prenatal complications	O16 Unspecified maternal hypertension
O20	Prenatal complications	O20 Haemorrhage in early pregnancy
O21	Prenatal complications	O21 Excessive vomiting in pregnancy
O22	Prenatal complications	O22 Venous complications and haemorrhoids in pregnancy
O23	Prenatal complications	O23 Infections of genitourinary tract in pregnancy
O24	Prenatal complications	O24 Diabetes mellitus in pregnancy
O25	Prenatal complications	O25 Malnutrition in pregnancy
O26	Prenatal complications	O26 Maternal care for other conditions predominantly related to pregnancy
O28	Prenatal complications	O28 Abnormal findings on antenatal screening of mother
O29	Prenatal complications	O29 Complications of anaesthesia during pregnancy
O30	Prenatal complications	O30 Multiple gestation
O31	Prenatal complications	O31 Complications specific to multiple gestation
O32	Prenatal complications	O32 Maternal care for known or suspected malpresentation of fetus
O33	Prenatal complications	O33 Maternal care for known or suspected disproportion
O34	Prenatal complications	O34 Maternal care for known or suspected abnormality of pelvic organs
O35	Prenatal complications	O35 Maternal care for known or suspected fetal abnormality and damage
O36	Prenatal complications	O36 Maternal care for other known or suspected fetal problems
O40	Prenatal complications	O40 Polyhydramnios
O41	Prenatal complications	O41 Other disorders of amniotic fluid and membranes
O42	Complicated delivery/perinatal care	O42 Premature rupture of membranes
O43	Prenatal complications	O43 Placental disorders
O44	Prenatal complications	O44 Placenta previa
O45	Prenatal complications	O45 Premature separation of placenta [abruptio placentae]
O46	Prenatal complications	O46 Antepartum haemorrhage, not elsewhere classified
O47	Uncomplicated prenatal care	O47 False labour
O48	Prenatal complications	O48 Prolonged pregnancy
O60	Complicated delivery/perinatal care	O60 Preterm labour and delivery
O61	Complicated delivery/perinatal care	O61 Failed induction of labour
O62	Complicated delivery/perinatal care	O62 Abnormalities of forces of labour
O63	Complicated delivery/perinatal care	O63 Long labour
O64	Complicated delivery/perinatal care	O64 Obstructed labour due to malposition and malpresentation of fetus
O65	Complicated delivery/perinatal care	O65 Obstructed labour due to maternal pelvic abnormality
O66	Complicated delivery/perinatal care	O66 Other obstructed labour
O67	Complicated delivery/perinatal care	O67 Labour and delivery complicated by intrapartum haemorrhage, not elsewhere classified
O68	Complicated delivery/perinatal care	O68 Labour and delivery complicated by fetal stress [distress]
O69	Complicated delivery/perinatal care	O69 Labour and delivery complicated by umbilical cord complications
O70	Complicated delivery/perinatal care	O70 Perineal laceration during delivery
O71	Complicated delivery/perinatal care	O71 Other obstetric trauma
O72	Complicated postnatal care	O72 Postpartum haemorrhage
O73	Complicated delivery/perinatal care	O73 Retained placenta and membranes, without haemorrhage
O74	Complicated delivery/perinatal care	O74 Complications of anaesthesia during labour and delivery

1	Prenatal complications
2	Complicated delivery/perinatal care
3	Uncomplicated prenatal care
4	Complicated postnatal care
5	Uncomplicated delivery
6	Uncomplicated postnatal care

O75	Complicated delivery/perinatal care	O75 Other complications of labour and delivery, not elsewhere classified
O80	Uncomplicated delivery	O80 Single spontaneous delivery
O81	Complicated delivery/perinatal care	O81 Single delivery by forceps and vacuum extractor
O82	Complicated delivery/perinatal care	O82 Single delivery by caesarean section
O83	Complicated delivery/perinatal care	O83 Other assisted single delivery
O84	Complicated delivery/perinatal care	O84 Multiple delivery
O85	Complicated postnatal care	O85 Puerperal sepsis
O86	Complicated postnatal care	O86 Other puerperal infections
O87	Complicated postnatal care	O87 Venous complications and haemorrhoids in the puerperium
O88	Complicated delivery/perinatal care	O88 Obstetric embolism
O89	Complicated postnatal care	O89 Complications of anaesthesia during the puerperium
O90	Complicated postnatal care	O90 Complications of the puerperium, not elsewhere classified
O91	Complicated postnatal care	O91 Infections of breast associated with childbirth
O92	Complicated postnatal care	O92 Other disorders of breast and lactation associated with childbirth
O94	Complicated postnatal care	O94 Sequelae of complication of pregnancy, childbirth and the puerperium
O97	Complicated postnatal care	O97 Death from sequelae of obstetric causes
O98	Prenatal complications	O98 Maternal infectious and parasitic diseases classifiable elsewhere but complicating pregnancy, childbirth and the pue
O99	Prenatal complications	O99 Other maternal diseases classifiable elsewhere but complicating pregnancy, childbirth and the puerperium
Z32	Uncomplicated prenatal care	Z32 Pregnancy examination and test
Z33	Prenatal complications	Z33 Pregnant state, incidental
Z34	Uncomplicated prenatal care	Z34 Supervision of normal pregnancy
Z35	Prenatal complications	Z35 Supervision of high-risk pregnancy
Z36	Uncomplicated prenatal care	Z36 Antenatal screening
Z37	Uncomplicated delivery	Z37 Outcome of delivery
Z38	Uncomplicated delivery	Z38 Liveborn infants according to place of birth
Z39	Uncomplicated postnatal care	Z39 Postpartum care and examination

ICD10 codes used in non-capitation based reimburesement (outpatient setting)

O10	Prenatal complications	O10 Pre-existing hypertension complicating pregnancy, childbirth and the puerperium
O11	Prenatal complications	O11 Pre-eclampsia superimposed on chronic hypertension
O12	Prenatal complications	O12 Gestational [pregnancy-induced] oedema and proteinuria without hypertension
O13	Prenatal complications	O13 Gestational [pregnancy-induced] hypertension
O14	Prenatal complications	O14 Pre-eclampsia
O15	Prenatal complications	O15 Eclampsia
O16	Prenatal complications	O16 Unspecified maternal hypertension
O20	Prenatal complications	O20 Haemorrhage in early pregnancy
O21	Prenatal complications	O21 Excessive vomiting in pregnancy
O22	Prenatal complications	O22 Venous complications and haemorrhoids in pregnancy
O23	Prenatal complications	O23 Infections of genitourinary tract in pregnancy
O24	Prenatal complications	O24 Diabetes mellitus in pregnancy
O25	Prenatal complications	O25 Malnutrition in pregnancy
O26	Prenatal complications	O26 Maternal care for other conditions predominantly related to pregnancy
O28	Prenatal complications	O28 Abnormal findings on antenatal screening of mother
O30	Prenatal complications	O30 Multiple gestation
O31	Prenatal complications	O31 Complications specific to multiple gestation
O32	Prenatal complications	O32 Maternal care for known or suspected malpresentation of fetus
O33	Prenatal complications	O33 Maternal care for known or suspected disproportion
O34	Prenatal complications	O34 Maternal care for known or suspected abnormality of pelvic organs
O35	Prenatal complications	O35 Maternal care for known or suspected fetal abnormality and damage
O36	Prenatal complications	O36 Maternal care for other known or suspected fetal problems
O40	Prenatal complications	O40 Polyhydramnios
O41	Prenatal complications	O41 Other disorders of amniotic fluid and membranes
O42	Complicated delivery/perinatal care	O42 Premature rupture of membranes
O43	Prenatal complications	O43 Placental disorders
O44	Prenatal complications	O44 Placenta previa
O46	Prenatal complications	O46 Antepartum haemorrhage, not elsewhere classified
O47	Uncomplicated prenatal care	O47 False labour
O48	Prenatal complications	O48 Prolonged pregnancy
O60	Complicated delivery/perinatal care	O60 Preterm labour and delivery
O61	Complicated delivery/perinatal care	O61 Failed induction of labour
O62	Complicated delivery/perinatal care	O62 Abnormalities of forces of labour
O63	Complicated delivery/perinatal care	O63 Long labour
O64	Complicated delivery/perinatal care	O64 Obstructed labour due to malposition and malpresentation of fetus
O65	Complicated delivery/perinatal care	O65 Obstructed labour due to maternal pelvic abnormality
O66	Complicated delivery/perinatal care	O66 Other obstructed labour
O67	Complicated delivery/perinatal care	O67 Labour and delivery complicated by intrapartum haemorrhage, not elsewhere classified
O68	Complicated delivery/perinatal care	O68 Labour and delivery complicated by fetal stress [distress]
O69	Complicated delivery/perinatal care	O69 Labour and delivery complicated by umbilical cord complications
O70	Complicated delivery/perinatal care	O70 Perineal laceration during delivery
O71	Complicated delivery/perinatal care	O71 Other obstetric trauma
O72	Complicated postnatal care	O72 Postpartum haemorrhage
O73	Complicated delivery/perinatal care	O73 Retained placenta and membranes, without haemorrhage
O75	Complicated delivery/perinatal care	O75 Other complications of labour and delivery, not elsewhere classified
O80	Uncomplicated delivery	O80 Single spontaneous delivery
O81	Complicated delivery/perinatal care	O81 Single delivery by forceps and vacuum extractor
O82	Complicated delivery/perinatal care	O82 Single delivery by caesarean section

1	Prenatal complications
2	Complicated delivery/perinatal care
3	Uncomplicated prenatal care
4	Complicated postnatal care
5	Uncomplicated delivery
6	Uncomplicated postnatal care

O83	Complicated delivery/perinatal care	O83 Other assisted single delivery
O84	Complicated delivery/perinatal care	O84 Multiple delivery
O85	Complicated postnatal care	O85 Puerperal sepsis
O86	Complicated postnatal care	O86 Other puerperal infections
O87	Complicated postnatal care	O87 Venous complications and haemorrhoids in the puerperium
O89	Complicated postnatal care	O89 Complications of anaesthesia during the puerperium
Z32	Uncomplicated prenatal care	Z32 Pregnancy examination and test
Z33	Prenatal complications	Z33 Pregnant state, incidental
Z34	Uncomplicated prenatal care	Z34 Supervision of normal pregnancy
Z35	Prenatal complications	Z35 Supervision of high-risk pregnancy
Z36	Uncomplicated prenatal care	Z36 Antenatal screening
Z37	Uncomplicated delivery	Z37 Outcome of delivery
Z38	Uncomplicated delivery	Z38 Liveborn infants according to place of birth
Z39	Uncomplicated postnatal care	Z39 Postpartum care and examination

ICD10 codes used in FKRTL reimburesement (inpatient setting)

1	Prenatal complications
2	Complicated delivery/perinatal care
3	Uncomplicated prenatal care
4	Complicated postnatal care
5	Uncomplicated delivery
6	Uncomplicated postnatal care

O10	Prenatal complications	O10 Pre-existing hypertension complicating pregnancy, childbirth and the puerperium
O11	Prenatal complications	O11 Pre-eclampsia superimposed on chronic hypertension
O12	Prenatal complications	O12 Gestational [pregnancy-induced] oedema and proteinuria without hypertension
O13	Prenatal complications	O13 Gestational [pregnancy-induced] hypertension
O14	Prenatal complications	O14 Pre-eclampsia
O15	Prenatal complications	O15 Eclampsia
O16	Prenatal complications	O16 Unspecified maternal hypertension
O20	Prenatal complications	O20 Haemorrhage in early pregnancy
O21	Prenatal complications	O21 Excessive vomiting in pregnancy
O22	Prenatal complications	O22 Venous complications and haemorrhoids in pregnancy
O23	Prenatal complications	O23 Infections of genitourinary tract in pregnancy
O24	Prenatal complications	O24 Diabetes mellitus in pregnancy
O25	Prenatal complications	O25 Malnutrition in pregnancy
O26	Prenatal complications	O26 Maternal care for other conditions predominantly related to pregnancy
O28	Prenatal complications	O28 Abnormal findings on antenatal screening of mother
O29	Prenatal complications	O29 Complications of anaesthesia during pregnancy
O30	Prenatal complications	O30 Multiple gestation
O31	Prenatal complications	O31 Complications specific to multiple gestation
O32	Prenatal complications	O32 Maternal care for known or suspected malpresentation of fetus
O33	Prenatal complications	O33 Maternal care for known or suspected disproportion
O34	Prenatal complications	O34 Maternal care for known or suspected abnormality of pelvic organs
O35	Prenatal complications	O35 Maternal care for known or suspected fetal abnormality and damage
O36	Prenatal complications	O36 Maternal care for other known or suspected fetal problems
O40	Prenatal complications	O40 Polyhydramnios
O41	Prenatal complications	O41 Other disorders of amniotic fluid and membranes
O42	Complicated delivery/perinatal care	O42 Premature rupture of membranes
O43	Prenatal complications	O43 Placental disorders
O44	Prenatal complications	O44 Placenta praevia
O45	Prenatal complications	O45 Premature separation of placenta [abruptio placentae]
O46	Prenatal complications	O46 Antepartum haemorrhage, not elsewhere classified
O47	Uncomplicated prenatal care	O47 False labour
O48	Prenatal complications	O48 Prolonged pregnancy
O60	Complicated delivery/perinatal care	O60 Preterm labour and delivery
O61	Complicated delivery/perinatal care	O61 Failed induction of labour
O62	Complicated delivery/perinatal care	O62 Abnormalities of forces of labour
O63	Complicated delivery/perinatal care	O63 Long labour
O64	Complicated delivery/perinatal care	O64 Obstructed labour due to malposition and malpresentation of fetus
O65	Complicated delivery/perinatal care	O65 Obstructed labour due to maternal pelvic abnormality
O66	Complicated delivery/perinatal care	O66 Other obstructed labour
O67	Complicated delivery/perinatal care	O67 Labour and delivery complicated by intrapartum haemorrhage, not elsewhere classified
O68	Complicated delivery/perinatal care	O68 Labour and delivery complicated by fetal stress [distress]
O69	Complicated delivery/perinatal care	O69 Labour and delivery complicated by umbilical cord complications
O70	Complicated delivery/perinatal care	O70 Perineal laceration during delivery
O71	Complicated delivery/perinatal care	O71 Other obstetric trauma
O72	Complicated postnatal care	O72 Postpartum haemorrhage
O73	Complicated delivery/perinatal care	O73 Retained placenta and membranes, without haemorrhage
O74	Complicated delivery/perinatal care	O74 Complications of anaethesia during labour and delivery

O75	Complicated delivery/perinatal care	O75 Other complications of labour and delivery, not elsewhere classified
O80	Uncomplicated delivery	O80 Single spontaneous delivery
O81	Complicated delivery/perinatal care	O81 Single delivery by forceps and vacuum extractor
O82	Complicated delivery/perinatal care	O82 Single delivery by caesarean section
O83	Complicated delivery/perinatal care	O83 Other assisted single delivery
O84	Complicated delivery/perinatal care	O84 Multiple delivery
O85	Complicated postnatal care	O85 Puerperal sepsis
O86	Complicated postnatal care	O86 Other puerperal infections
O87	Complicated postnatal care	O87 Venous complications and haemorrhoids in the puerperium
O88	Complicated delivery/perinatal care	O88 Obstetric embolism
O89	Complicated postnatal care	O89 Complications of anaethesia during puerperium
O90	Complicated postnatal care	O90 Complications of the puerperium, not elsewhere classified
O91	Complicated postnatal care	O91 Infections of breast associated with childbirth
O92	Complicated postnatal care	O92 Other disorders of breast and lactation associated with childbirth
O94	Complicated postnatal care	O94 Sequelae of complications of pregnancy, childbirht and puerperium
O95	Complicated postnatal care	O95 Obstetric death of unspecified cause
O96	Complicated postnatal care	O96 Death from any obstetric cause occurring more than 42 days but less than one year after delivery
O98	Prenatal complications	O98 Maternal infectious and parasitic diseases classifiable elsewhere but complicating pregnancy, childbirth and the pue
O99	Prenatal complications	O99 Other maternal diseases classifiable elsewhere but complicating pregnancy, childbirth and the puerperium
Z32	Uncomplicated prenatal care	Z32 Pregnancy examination and test
Z33	Prenatal complications	Z33 Pregnant state, incidental
Z34	Uncomplicated prenatal care	Z34 Supervision of normal pregnancy
Z35	Prenatal complications	Z35 Supervision of high-risk pregnancy
Z36	Uncomplicated prenatal care	Z36 Antenatal screening
Z38	Uncomplicated delivery	Z38 Liveborn infants according to place of birth
Z37	Uncomplicated delivery	Z37 Outcome of delivery
Z39	Uncomplicated postnatal care	Z39 Postpartum care and examination
