# Supplementary material for: Obstetrics care in Indonesia: Determinants of maternal mortality and stillbirth rates
Source: PLoS One. 2024 Jul 5;19(7):e0303590. doi: 10.1371/journal.pone.0303590 (PMC11226051; doi:10.1371/journal.pone.0303590)

Obstetrics care in Indonesia : Determinants of

maternal mortality and stillbirth rates

Supplementary file 3

Suppl. Figure S1: Quadrant charts for usage of PHC structures under capitation setting (FKTP) A) compared to FKRTL usage; and for complicated diagnoses associated with relative numbers of rural population. B) antenatal/rural population; C) perinatal/rural population; D) postnatal/rural population.

A B


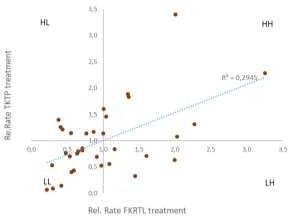

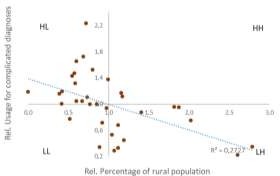


C


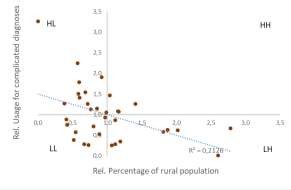

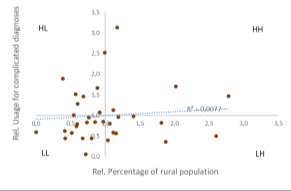


Suppl. Figure S2: Quadrant charts for FKTP usage of PHC structures under capitation setting for complicated diagnoses associated with relative availability of human resources. A) all PHC treatments/all midwifes; B) all PHC treatments/ midwifes in 3T regions; inpatient treatment associated with relative numbers of C) overall and D) 3T availability of midwifes, E) SPOG (overall) and F) rel. part of rural population within each province.


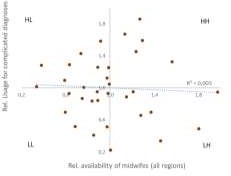

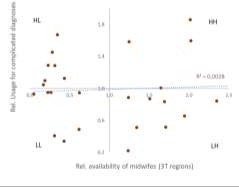
A B

C D


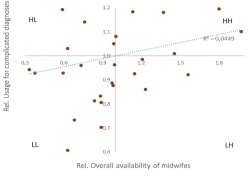

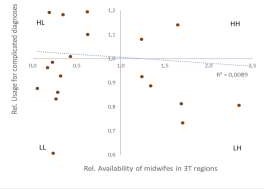


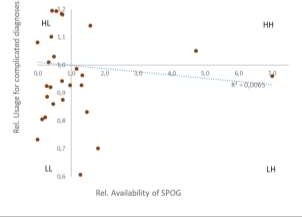

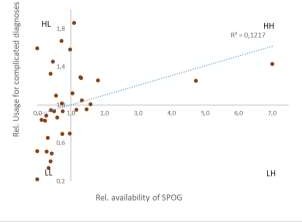
E F

Suppl. Figure S3: Quadrant charts for outcome related to relative usage of obstetrics primary (A; B) and advanced care (C; D) to stillbirth rates (A; C) and maternal death rates (B; D).

A B


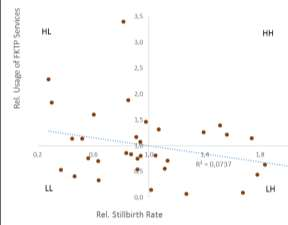

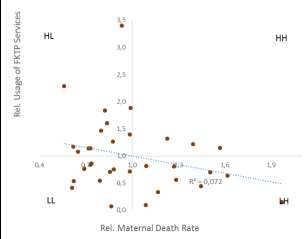


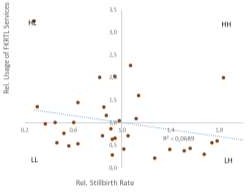

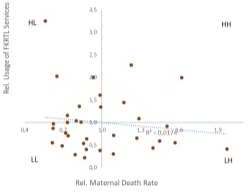
C D

Suppl. Figure S4: Quadrant charts for outcome related to availability of gynecologists without outlayer (Provinces Bali and Jakarta) to stillbirth rates (A) and maternal death rates (B). C) Number of C-sections related to the number of available SPOG; D) C-section rate compared to population based availability of SPOG; E) Maternal death rate related C-section rates; C-section rate related to C- section/SPOG ratio

A B


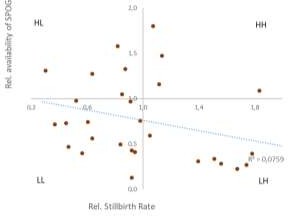

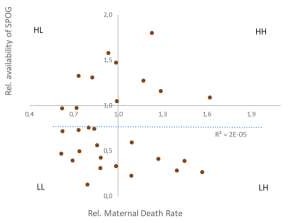


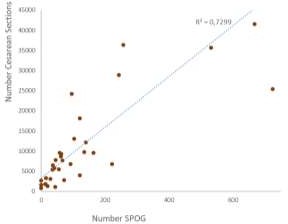

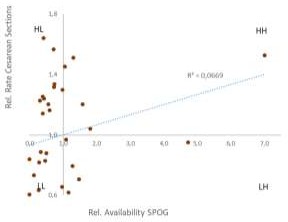
C D

E F


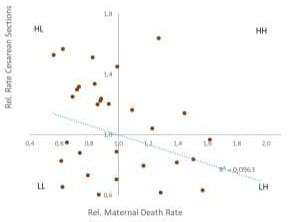

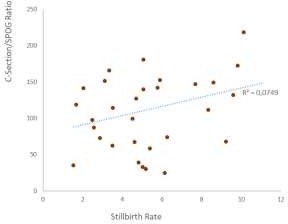

Supplement: S3 File — (DOCX) [file pone.0303590.s003.docx]
