# Supplementary figures and images for: Obstetrics care in Indonesia: Determinants of maternal mortality and stillbirth rates
Source: PLoS One. 2024 Jul 5;19(7):e0303590. doi: 10.1371/journal.pone.0303590 (PMC11226051; doi:10.1371/journal.pone.0303590)

Supplementary file 4

| A | B |
| --- | --- |
| 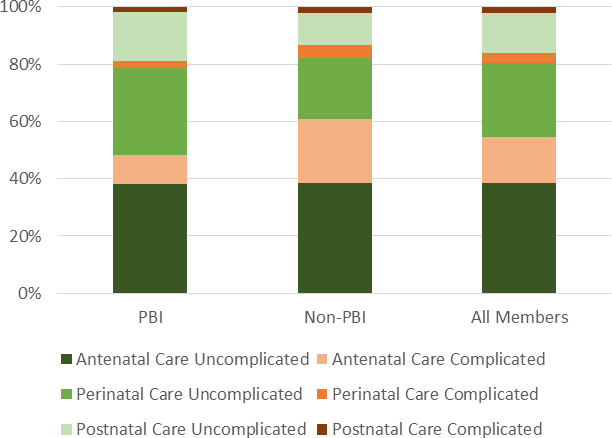 | 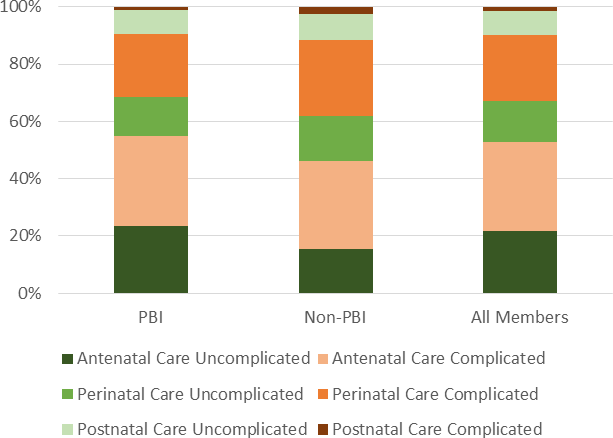 |
| C | D |
| 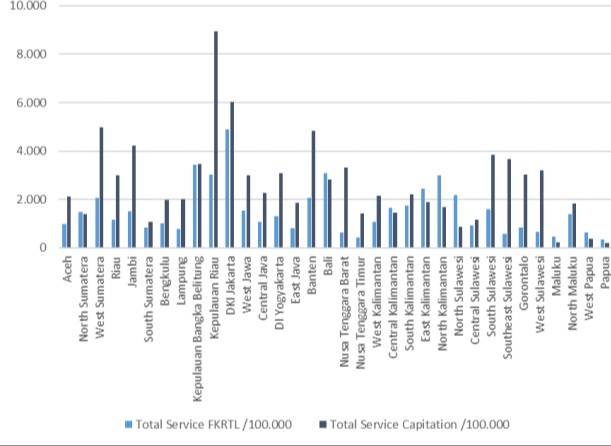 | 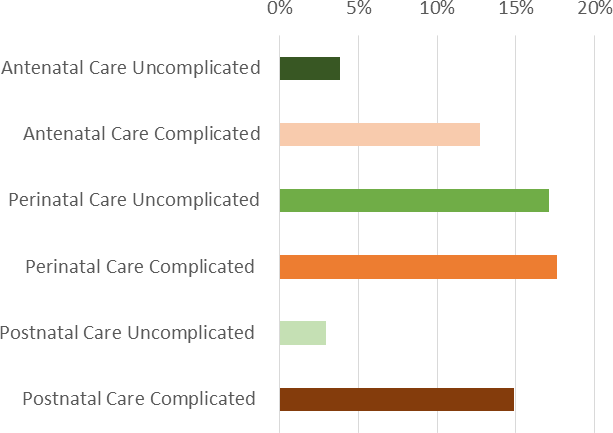 |
| E | F |
| 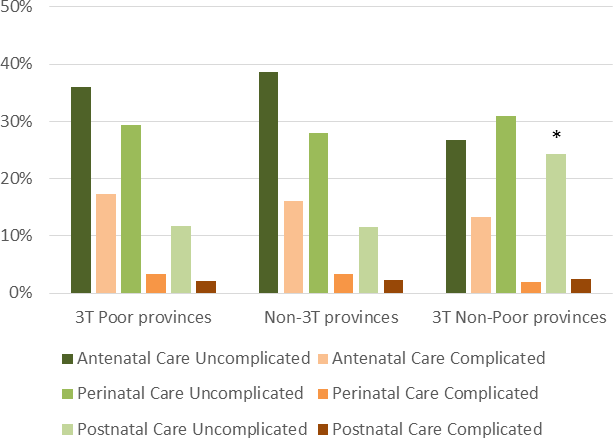 | 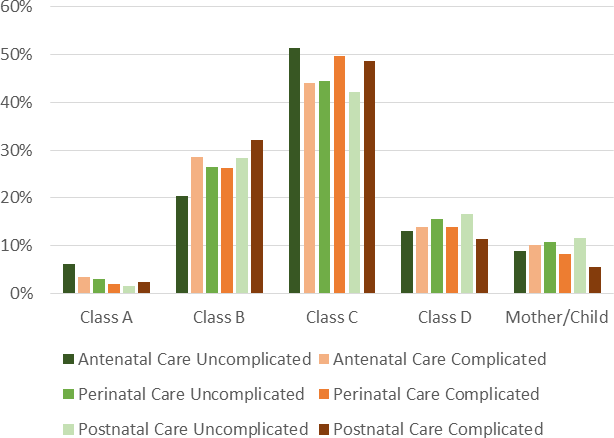 |

| A | B |
| --- | --- |
| 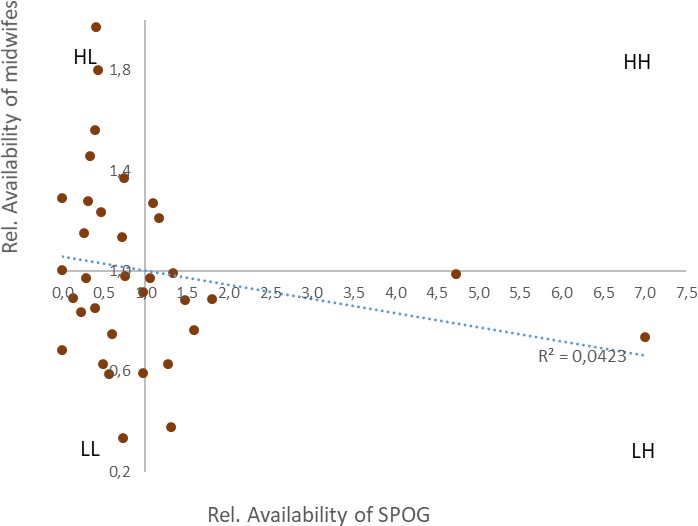 | 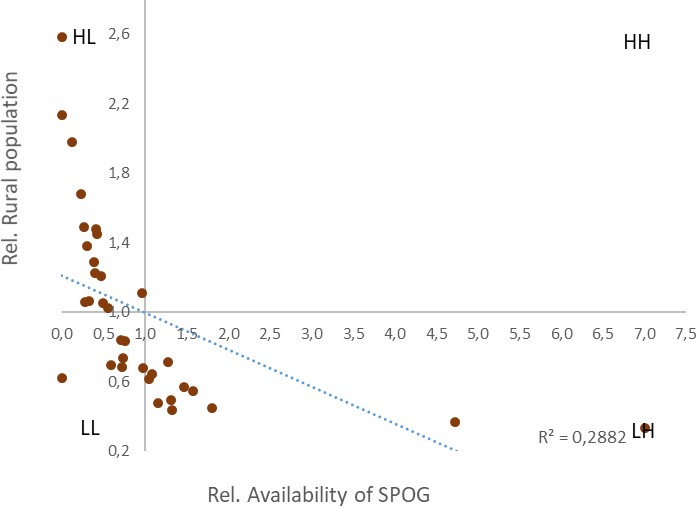 |
| C | D |
| 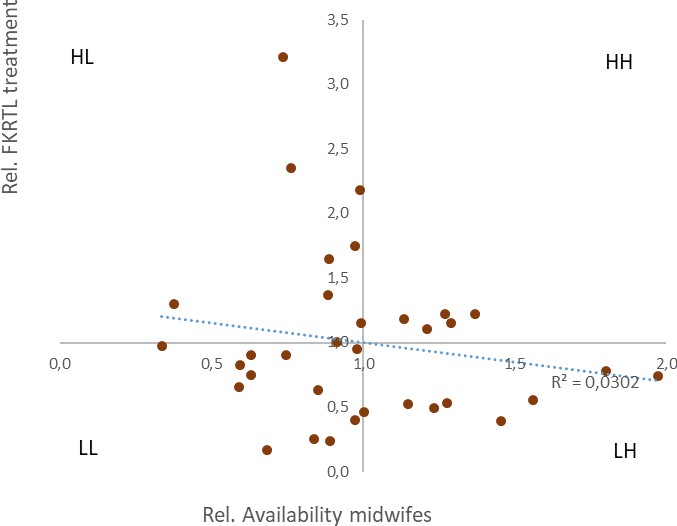 | 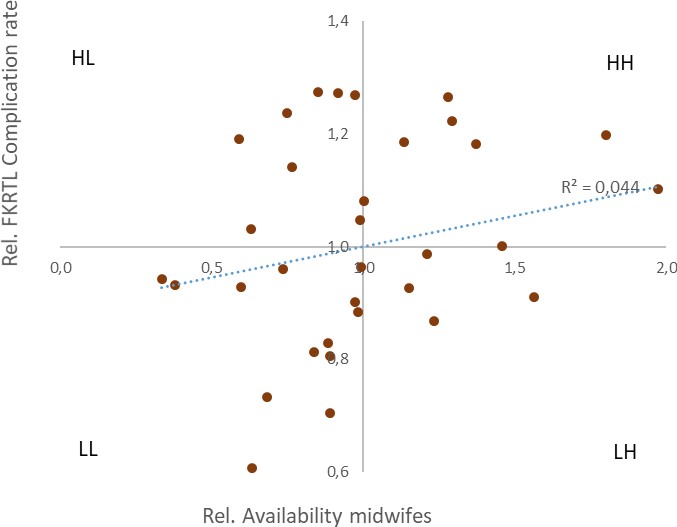 |
| E | F |
| 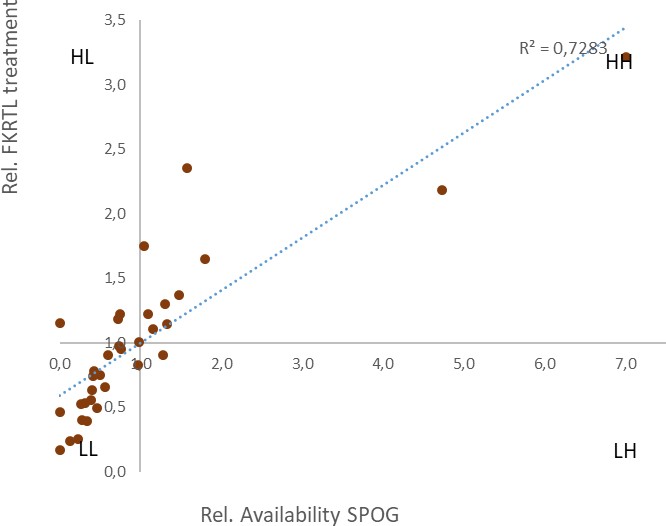 | 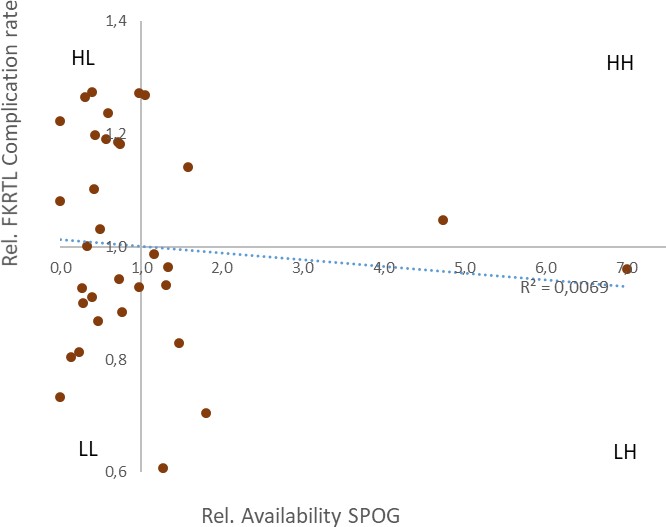 |

| A | B |
| --- | --- |
| 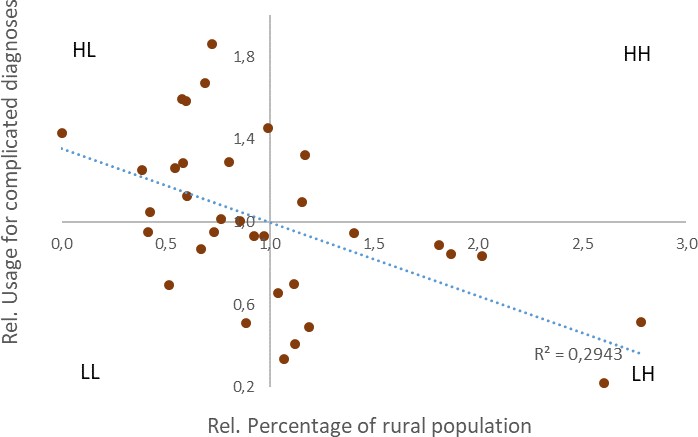 | 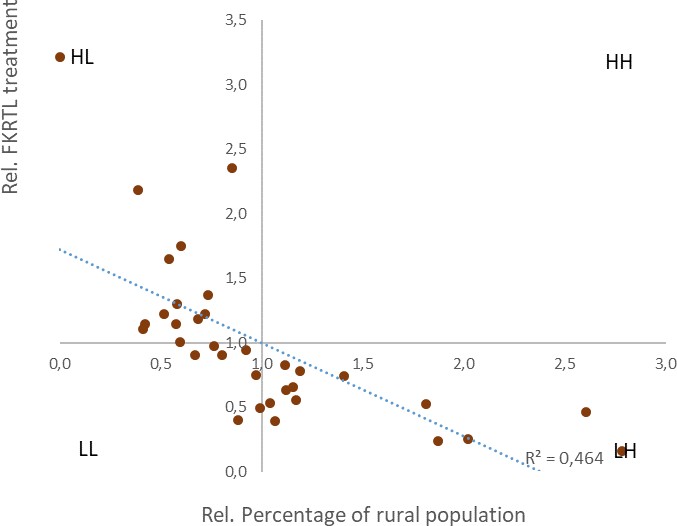 |
| C |  |
| 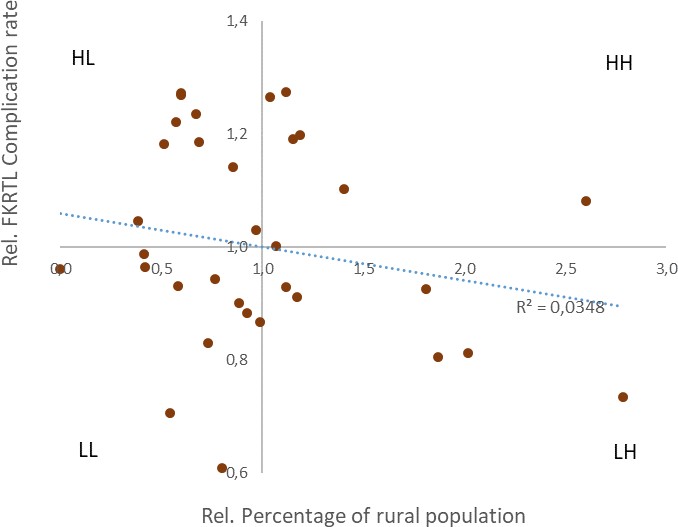 |  |

| A | B |
| --- | --- |
| 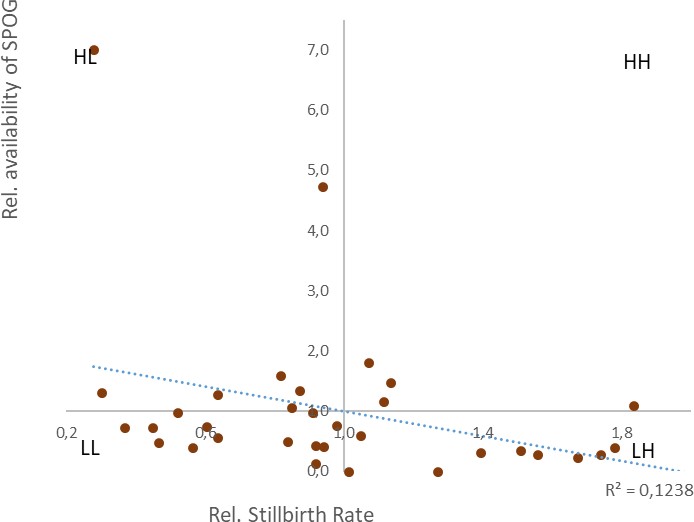 | 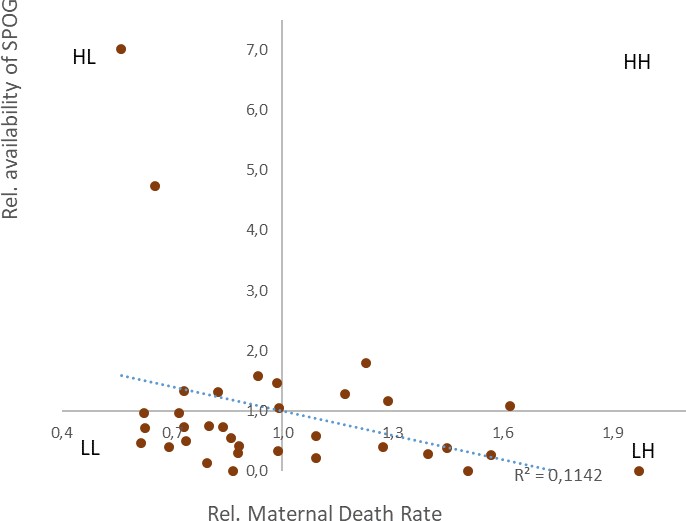 |
| C | D |
| 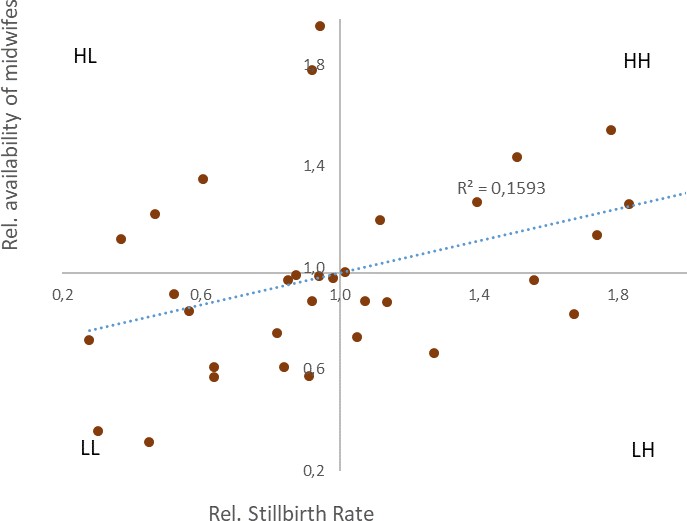 | 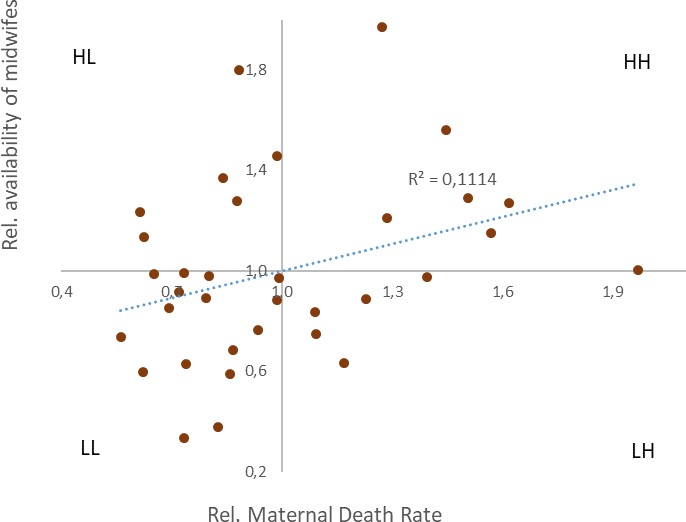 |
| E | F |
| 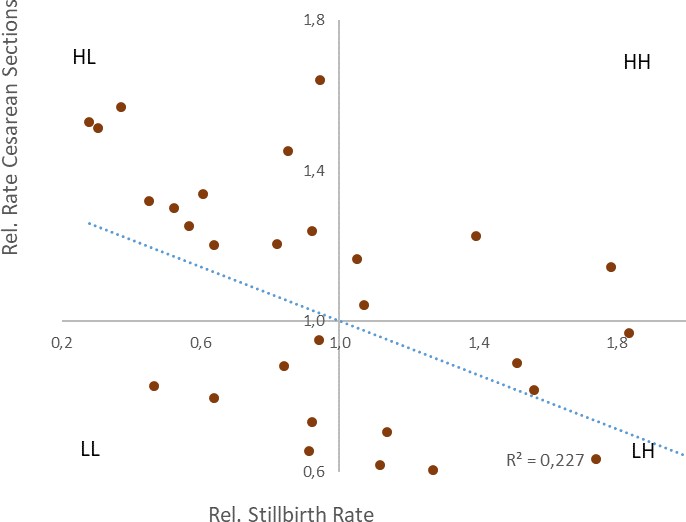 | 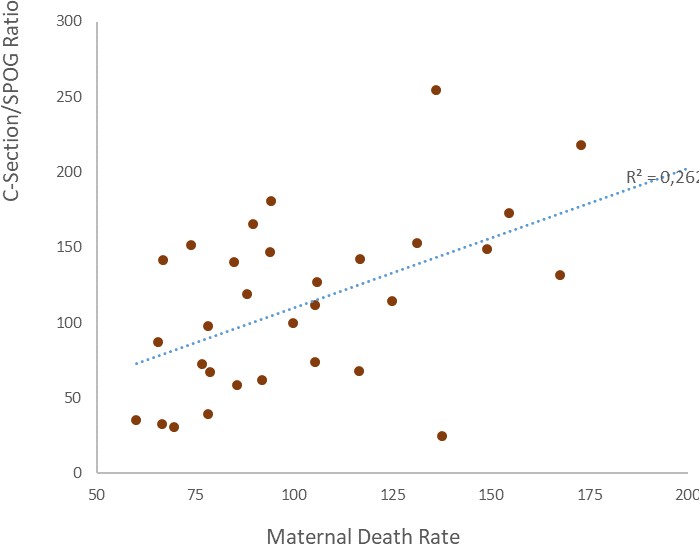 |

Supplement: S4 File — (DOCX) [file pone.0303590.s004.docx]
