# Supplementary material for: Obstetrics care in Indonesia: Determinants of maternal mortality and stillbirth rates
Source: PLoS One. 2024 Jul 5;19(7):e0303590. doi: 10.1371/journal.pone.0303590 (PMC11226051; doi:10.1371/journal.pone.0303590)
Supplement: Attachment — Submitted filename: Response to reviewer-2024.docx [file pone.0303590.s005.docx]

Response to reviewer

Editor

1. Kindly consider revising the Title of your manuscript to better reflect what was included in this study and reported in the manuscript.

2. Kindly revise your abstract to include research objectives, statistical methods, including sampling methodology, results and conclusions derived from the study.

3. Kindly revise your introduction to provide a more in-depth background to the study including research objectives and the aims of this particular study. Also, fully explain unclear abbreviations the first time used, before using abbreviations in the rest of the manuscript. If necessary you may include a table of abbreviations at the end of the manuscript.

4. In the methods section kindly revise and better explain the statistical approach and parameters used in the study, including sampling methodology.

5. In the Results section kindly revise to better explain the findings from the study and its interpretation.

6. Kindly revise your discussion in the light of the findings from your own study in comparison to other recent and similar studies in Indonesia, and elsewhere in the literature.

7. Insert a Statement Limitations regarding the findings from this study and their potential for generalization and areas for future study.

8. The authors may consider using an English language editor to address all the grammatical and typographical errors in the manuscript.

8. Kindly address all other comments and observations as outlined by the peer-reviewers.

Answer

Thank you for the excellent revisions to this manuscript.

1. The title has been corrected to Obstetrics care in Indonesia: Determinants of maternal mortality and stillbirth rates
2. The abstract has been revised according to input
3. The introduction has been made complete with research objectives and added abbreviations at the end
4. The method has been revised by adding a statistical approach and parameters used in the study, including sampling methodology.
5. The results section has been given a more detailed interpretation
6. The discussion is equipped with references from several countries, especially Indonesia
7. Study limitations and further research that can be developed from this study have been given
8. grammatical and typographical errors in the manuscript have been revised

Reviewer 1

1. edit one reference citation (Methods section, 2nd paragraph);
2. mention the classic assumptions need to be met for linear regression and/or ANOVA. One of them is diagnostic test of the residuals and mention the criteria for choosing the best model;
3. use of the coefficient of correlation instead of the coefficient of determination in explaining bivariate correlation.
4. mention more specific the independent and dependent variables in their research;
5. mention the reason of the choice of those variables in point 4.
6. explain why the independent variables for each outcome variable in this research are different.
7. further discuss the connection between the results of this study with the previous studies.
8. review the writing style, especially when using fullstop (.) before refering a certain figure or table (example: Results section, 1st paragraph, 2nd sentence).
9. write one paragraph consists of minimum three sentences.

Answer

Thank you for the excellent revisions to this manuscript

1. Citations have been edited

2. Modelling criteria were more extensively described in the methods section.

3. Correlation coefficients (non-standardized and standardized) including their confidence intervals are provided in table 1

4. Is added

5. Is added

6. In the beginning we used the same set of variables for both analyses, but during the stepwise procedures the obtained relevant variables were slightly different for the two outcome targets

7. We extended this topic in the discussion
8. Is modified

Reviewer 2

This study presents an overview of obstetric care in Indonesia in terms of the use of health insurance organized by the Indonesian government, namely jaminan kesehatan nasional (JKN). The authors want to explain obstetric services in Indonesia based on this insurance scheme (JKN) in providing protection for maternal health services, especially in increasing access to obstetric services for low-income families with various challenges related to coverage and services.
• Although it was not explicitly explained regarding the novelty of this study, the authors explained that this study was slightly different from the previous studies by using national-scale dataset sample, namely from BPJS implementation for the 2014-2018 period to analyze the outcomes and utilization of maternal health services, as well as differences related to demographic and economic subgroup.
• However, there are still several principles of scientific writing that are not consistent and still receive less attention from a feasibility perspective. Important things that need to be considered and consistent start from identifying problems, developing research methods, analyzing and interpreting research results to drawing conclusions and provide recommendations to readers.
• Furthermore, to improve the quality of this article, it is recommended that the authors consult further with a biostatistician or epidemiologist. The authors should also pay more attention to the many sentences that are still not written correctly.

Authors

Answer

Thank you for the excellent revisions to this manuscript

1. The author has added information related to obstetric services in Indonesia based on this insurance scheme (JKN) in providing protection for maternal health services
2. The author has added novelty of this study
3. The article writing has been revised
4. The author has consulted with all team members to improve this article
